# Development and validation of a tumor size-stratified prognostic nomogram for patients with gastric signet ring cell carcinoma

**DOI:** 10.1007/s13304-024-02020-0

**Published:** 2024-11-04

**Authors:** Xin Hui, Guangbo Zhou, Ya Zheng, Yuping Wang, Qinghong Guo

**Affiliations:** 1 The First Clinical Medical College, Lanzhou University, Lanzhou, China; 2 Department of Gastroenterology, The First Hospital of Lanzhou University, No.1, Donggang West Road, Chengguan District, Lanzhou, 730000 Gansu Province China; 3 Gansu Province Clinical Research Center for Digestive Diseases, The First Hospital of Lanzhou University, No.1, Donggang West Road, Chengguan District, Lanzhou, 730000 Gansu Province China

**Keywords:** Gastric signet ring cell, Tumor size, Restricted cubic spline (RCS), Nomogram, Prognosis, SEER, Survival

## Abstract

Gastric signet ring cell carcinoma (GSRC) is a rare malignancy without a commonly acknowledged prognostic assessment and treatment system. This study aimed to determine the optimal cut-off value of tumor size (TS), and construct a prognostic nomogram in combination with other independent prognostic factors (PFs) to predict 3 year and 5 year overall survival (OS) in GSRC patients. From the Surveillance, Epidemiology, and End Results (SEER) database, this study collected 4744 patients diagnosed with GSRC. These patients were randomized into a training cohort (*n* = 2320,) and a validation cohort (*n* = 1142). A restricted cubic spline (RCS) was used to determine the cut-off value for TS, and univariate and multivariate Cox regression analyses were performed in the training cohort to identified significant predictors. A prognostic nomogram was constructed to predict OS at 3 and 5 years. Concordance index (C index), receiver operating characteristics curve (ROC curve), area under curve (AUC), and calibration curve were used to test the predictive accuracy of the model. A non-linear relationship was observed between TS and the risk of OS in GSRC, with TS thresholds at 4.4 cm and 9.6 cm. Survival was significantly lower in GSRC patients with TS > 4.4 cm. Age, marriage, chemotherapy, surgery, TS, SEER stage, regional lymph node status, and total number were independent predictors of OS. The C index in the training cohort was 0.748, and the AUC values for both 3- and 5-year OS were higher than 0.80. Similar results were observed in the validation cohort. In addition, the calibration curves showed good agreement between the predicted 3 year and 5 year OS and the actual OS. TS is a key prognostic factor for patients with GSRC, and patients with a TS of 4.4–9.6 cm and > 9.6 cm may have a poorer prognosis than those with a TS of < 4.4 cm. The TS-stratified nomogram we constructed and validated has favorable accuracy and calibration precision, and may be helpful in predicting the survival rate of patients.

## Introduction

Gastric cancer (GC) is one of the most prevalent malignant tumors worldwide. According to GLOBOCAN 2020 (Global Cancer Statistics 2020), among all cancers, GC ranks fifth in incidence and fourth in mortality among all cancers, next only to lung cancer and colorectal cancer [1]. Although the incidence of GC (intestinal type) has declined in recent years, thanks to the broad elimination of *Helicobacter pylori* and the early diagnosis of GC, the incidence of diffuse GC, especially gastric signet ring cell carcinoma (GSRC), has been increasing [2]. According to recent studies, SRCC accounts for 3.4–39% of cases of gastric adenocarcinoma [3, 4]. GSRC has unique clinicopathological features. In the WHO classification of GC histology, gastric adenocarcinoma is divided into papillary adenocarcinoma, mucinous adenocarcinoma, tubular adenocarcinoma, signet ring cell carcinoma (SRCC), and undifferentiated carcinoma. SRCC is defined as a malignant tumor composed entirely or predominantly of signet ring cells [5]. The cancer cells of SRCC are rich in mucin, which pushes the nucleus to the periphery of cells, forming a characteristic ring-like appearance [6]. According to the Japanese classification system, GSRC is classified as the undifferentiated type [7]. Lauren classification is the most widely used classification of GC. In the Lauren classification, SRCC is classified as a diffuse type [8]. GSRC is a highly malignant tumor characterized by poor differentiation, high invasiveness, and unfavorable prognosis [9, 10]. Tumor size (TS), an important clinical factor, has considerable prognostic value for many kinds of tumors, including GSRC. As one of the valuable clinicopathological features, TS has been extensively analyzed in many tumor staging systems, including the American Joint Committee on Cancer (AJCC) staging system. However, in these staging systems, the classification for T-staging of gastric cancer is based only on the depth of infiltration of the primary tumor and is not related to TS. In addition, the cut-off value of TS was determined through X-tile or median in most studies, where the relationship between the continuous variables and the patient survival was linear by default, ignoring the possible non-linear relationship between them. Therefore, the independent predictive value of TS for the survival outcome of GSRC patients has not yet been determined: the optimal cut-off value of TS remains to be determined, and it is unclear whether it can predict the overall survival (OS) of GSRC patients in combination with other prognostic factors (PFs). This paper aimed to investigate the independent predictive value of TS in GSRC patients with population-based data obtained from the SEER database, a U.S. cancer database covering nearly 48% of the US population, to explore the potential non-linear relationship between TS and prognosis of GSRC and explore the optimal cut-off value of TS using the restricted cubic spline (RCS) model, as well as construct a prognostic nomogram based on the COX proportional hazards model (COX model) to predict the OS of GSRC patients in combination with other PFs.

## Methods

### Research subjects and data extraction

All data analyzed in the current study were collected from the SEER database, which is supported by the U.S. National Cancer Institute and provides clinical data on cancer patients. All data extraction procedures in the present study were performed using SEER*Stat software (version 8.4.0.1, seer.cancer.gov/seerstat). Data on patients diagnosed with GSRC between 2004 and 2015 were collected. Inclusion criteria: (1) International Classification of Diseases, Third Edition (ICD-O-3), pathological type code 8490/3, and (2) primary GSRC diagnosed between 2004 and 2015. Exclusion criteria: (1) Data of the patients were unknown, including race, stage, grade, surgery, radiotherapy and chemotherapy, TS, and survival time; (2) The survival time is 0. The variables included in the statistical analysis were: demographics (age, sex, race, marriage), primary site, total number, TS, grade, SEER stage, surgery, radiotherapy and chemotherapy, regional lymph node status, survival time, and vital status. The endpoint of the study was OS, which was defined as the time period from diagnosis to death or the final follow-up. TS was evaluated comprehensively based on combined clinical and operative/pathological assessment. In the event of a discrepancy between pathology and operative reports, pathology report is given priority.

### Determination of the optimal cut-off value of TS

Statistically significant outcomes in the univariate Cox regression analysis were embedded as confounders in the RCS model with 4-node (5th, 25th, 65th, and 95th percentiles of TS) to explore the potential relationship between TS and the prognosis, and determine the optimal cut-off value of TS. Thereafter, stratified by TS, Kaplan–Meier curves (KM curves) were plotted, and the cumulative survival of different subgroups of patients was compared using the log-rank sum test. The variables with p < 0.05 in univariate COX regression analysis were included in multivariate COX regression analysis to analyze the predictive value of TS for OS in GSRC patients. The results were expressed as hazard ratio (HR) with 95% confidence interval (CI).

### Model construction

R software was employed to randomly assign all patients to the training cohort or validation cohort at a ratio of 7:3. Univariate and multivariate COX models were used to analyze the risk factors (RFs) associated with OS of GSRC patients. Variables, of which the P value was below 0.05 in the univariate COX model, were included in the multivariate COX model for further analysis. The independent predictors of OS were identified and used to construct the 3- and 5-year OS prognostic nomogram based on the results of multivariate COX regression analysis. In the validation, the concordance index (C index) was calculated to evaluate the discrimination ability of the nomogram. Sensitivity and specificity of the nomogram were assessed using the receiver operating characteristic curve (ROC) and area under the ROC curve (AUC), and a calibration curve was drawn to further assess the performance of the nomogram. Generally speaking, a C index and AUC values greater than 0.7 indicate a strong discrimination ability of the prediction tool.

### Statistical analysis

In this study, frequency and percentage were used to describe the categorical variables. Mean value ± standard deviation was used to describe the continuous variables. Chi-square test was adopted to compare the differences between categorical variables, and independent samples t test was adopted to compare the differences between continuous variables. R (version 4.2.2) is used for all statistical analyses. All tests were two-sided. A *P* value < 0.05 (two-sided) was of statistical significance.

## Results

### Baseline characteristics of the patients

A total of 4744 patients diagnosed with GSRC between 2004 and 2015 from the SEER database were included in this study. Randomly, seventy percent of the patients were assigned to the training cohort (*n* = 3320) and the remaining 30% to the validation cohort (*n* = 1424). No statistical differences were observed in baseline characteristics between the training set and the validation set (*P* > 0.05) (Table 1). Among all patients, there were slightly more male patients than female patients (*n* = 250,8, 52.9% vs *n* = 2236, 47.1%). The majority of patients were younger (< 65 years, *n* = 2690, 56.7%), married (*n* = 2858, 60.2%), and white (*n* = 3258, 68.7%) people. Patients with positive LNM were about twice as many as patients with negative LNM (*n* = 2320, 48.9%; *n* = 1142, 24.1%). Tumor grade III (*n* = 4009, 84.5%) was predominant, and most patients had regional (*n* = 2380, 50.2%) or distant metastasis (*n* = 1236, 26.1%). In addition, more than half of the patients received surgery (*n* = 3580, 75.5%) and chemotherapy (*n* = 2897, 61.1%), and most of the patients did not receive radiotherapy (*n* = 3137, 66.1%).

**Table 1:** Characteristics between the training cohort and the validation cohort

Characteristics	level	Overall	Training	Testing	*P *value
		4744	3320	1424	
Age (years), *n* (%)	< 65	2690 (56.7)	1897 (57.1)	793 (55.7)	0.372
	> = 65	2054 (43.3)	1423 (42.9)	631 (44.3)	
Marriage, *n* (%)	Unknown	1715 (36.2)	1214 (36.6)	501 (35.2)	0.574
	Married	2858 (60.2)	1984 (59.8)	874 (61.4)	
	Unmarried	171 (3.6)	122 (3.7)	49 (3.4)	
Sex, *n* (%)	Male	2508 (52.9)	1748 (52.7)	760 (53.4)	0.672
	Female	2236 (47.1)	1572 (47.3)	664 (46.6)	
Race, *n* (%)	White	3258 (68.7)	2259 (68.0)	999 (70.2)	0.16
	Other^a^	1486 (31.3)	1061 (32.0)	425 (29.8)	
Primary site, *n* (%)	proximal third^b^	1043 (22.0)	726 (21.9)	317 (22.3)	0.144
	middle^c^	1437 (30.3)	1037 (31.2)	400 (28.1)	
	distal third^d^	1417 (29.9)	982 (29.6)	435 (30.5)	
	Other^e^	847 (17.9)	575 (17.3)	272 (19.1)	
Grade, *n* (%)	I/II	130 (2.7)	97 (2.9)	33 (2.3)	0.602
	III	4009 (84.5)	2793 (84.1)	1216 (85.4)	
	IV	141 (3.0)	100 (3.0)	41 (2.9)	
	Unknown	464 (9.8)	330 (9.9)	134 (9.4)	
SEER stage, *n* (%)	Distant	1236 (26.1)	859 (25.9)	377 (26.5)	0.694
	Localized	1056 (22.3)	748 (22.5)	308 (21.6)	
	Regional	2380 (50.2)	1659 (50.0)	721 (50.6)	
	Unknown	72 (1.5)	54 (1.6)	18 (1.3)	
Surgery, *n* (%)	No	1164 (24.5)	806 (24.3)	358 (25.1)	0.551
	Yes	3580 (75.5)	2514 (75.7)	1066 (74.9)	
Radiation, *n* (%)	No	3137 (66.1)	2181 (65.7)	956 (67.1)	0.353
	Yes	1607 (33.9)	1139 (34.3)	468 (32.9)	
Chemotherapy, *n* (%)	No	1847 (38.9)	1287 (38.8)	560 (39.3)	0.741
	Yes	2897 (61.1)	2033 (61.2)	864 (60.7)	
Regional lymph node status, *n* (%)	Negative	1142 (24.1)	822 (24.8)	320 (22.5)	0.23
	Positive	2320 (48.9)	1605 (48.3)	715 (50.2)	
	Unknown	1282 (27.0)	893 (26.9)	389 (27.3)	
Total number, *n* (%)	1	4396 (92.7)	3072 (92.5)	1324 (93.0)	0.631
	> 1	348 (7.3)	248 (7.5)	100 (7.0)	
Tumor size (mm)	–	53.8 (54.5)	52.8 (50.2)	563 (63.3)	0.041
Vital status (%)	Alive	1162(24.5)	823 (24.8)	339 (23.8)	0.494
	Dead	3582 (75.5)	2497 (75.2)	1085 (76.2)	
Survival time (month)	–	37.9(42.7)	38.3 (43.1)	36.9 (42.0)	0.277

SEER stage, SEER stage is defined by the SEER database referred to as the SEER stage in our article that provides information about each cancer (primary site/histology/other factors defined) schema

*SEER* surveillance, epidemiology, and end results program

^a^Other, American Indian/Alaska Native/Asian or Pacific Islander/Black/Unknown;

^b^proximal third, cardia/fundus;

^c^middle, body/lesser curvature /greater curvature;

^d^distal third, antrum/pylorus;

^e^Other, NOS/overlapping lesion;

### Optimal cut-off value of TS

**Fig. 1:**
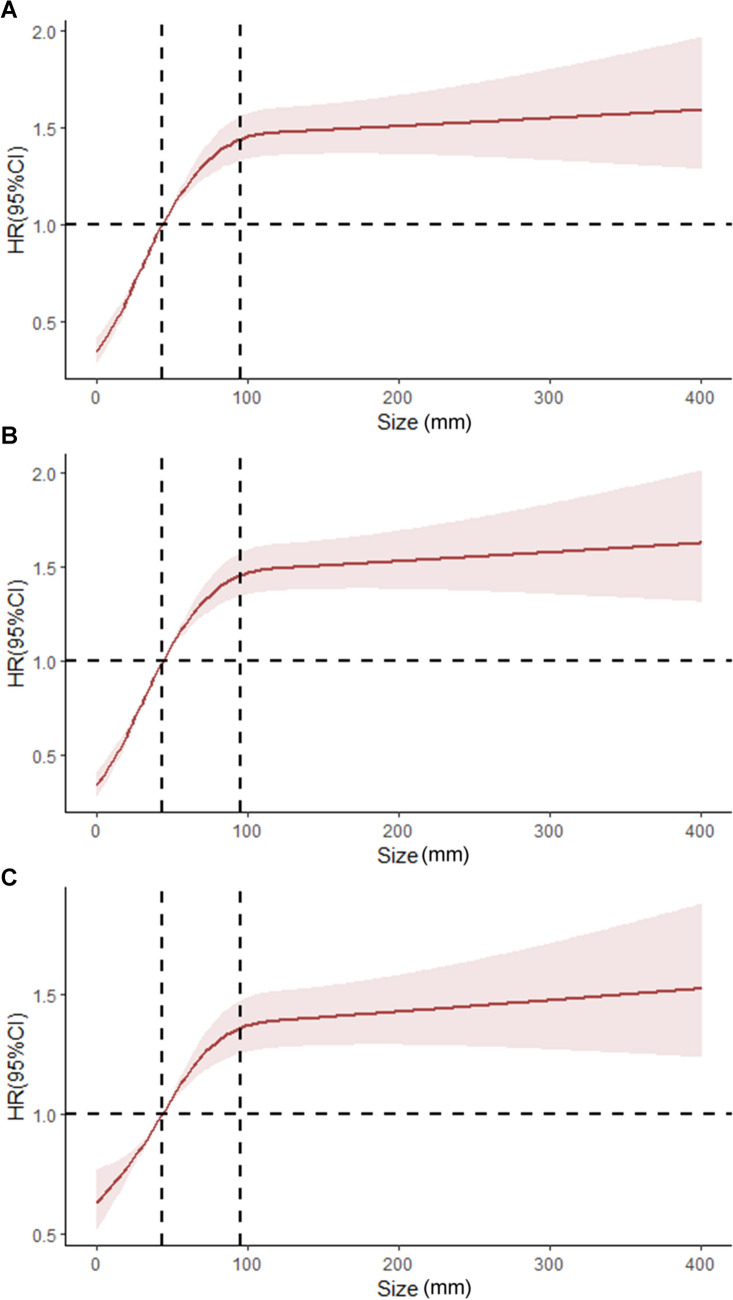
RCS model of tumor size and HR in patients with GSRC. **A** Adjusted for nothing; **B** Adjusted for age, race, and marriage; **C** Adjusted for age, race, marriage, primary site, grade, stage, surgery, radiotherapy and chemotherapy, regional lymph node status, and the number of tumors. *RCS* restricted cubic spline; *GSRC* gastric signet ring cell carcinoma; *HR* hazard ratio; *95% CI* 95% confidence interval; Tumor size (mm)

RCS adjusted for all confounding factors showed a non-linear relationship between TS and OS in GSRC patients, with the thresholds of TS at 4.4 cm and 9.6 cm. As TS increased, the risk of death in patients elevated accordingly. When TS was 4.4 cm, the corresponding HR was close to 1. As TS continued to increase in the range of 4.4–9.6 cm, the risk of mortality in patients was significantly higher and kept rising. When TS exceeded 9.6 cm, the risk of mortality gradually plateaued out, but still remained at a high level, as shown in Fig. 1 (A, B, and C). Patients were divided into 3 groups according to the thresholds: < 4.4 cm, 4.4–9.6 cm, and > 9.6 cm.

### Tumor characteristics and survival probability of GSRC patients with different tumor sizes

**Fig. 2:**
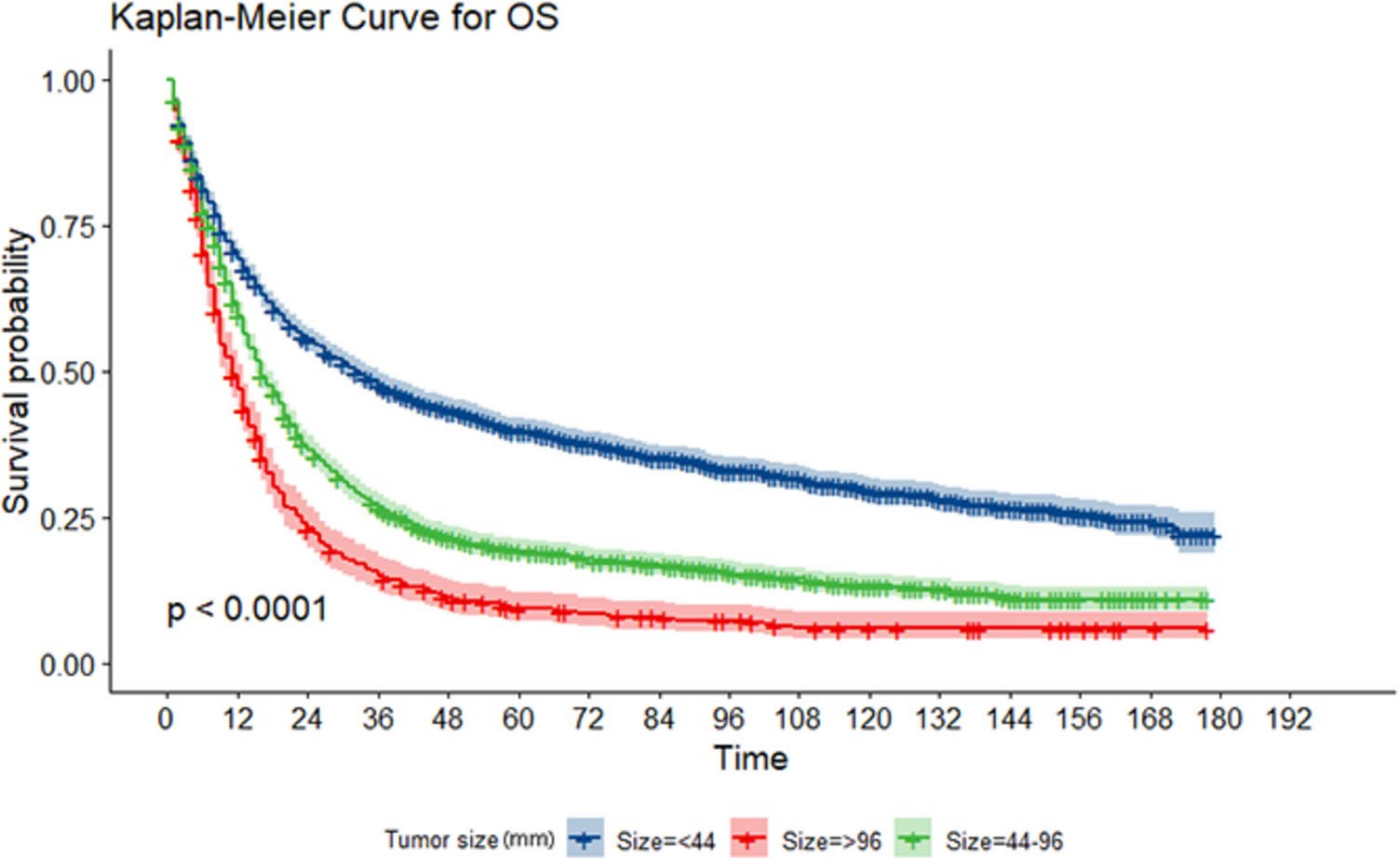
Kaplan–Meier curves for cumulative survival of GSRC patients. Time (month); Tumor size (mm); *GSRC* gastric signet ring cell carcinoma

To further demonstrate clinical significance of this threshold for TS, we compared the variations in tumor characteristics across the three groups. Statistically significant differences were observed in sex, primary site, grade, stage, surgery, radiotherapy and chemotherapy, lymph node metastasis (LNM) status, and total number of primary tumors between the three groups. According to our analysis, the larger TS group was more likely to be male patients, have a tumor at distal third, develop distant metastasis, have positive local lymph nodes, and undergo surgical resection and chemotherapy (all *P* < 0.05) (Table 2). The KM survival curves showed a significant difference in the cumulative survival rate among the three groups (*P* < 0.001). As shown in Fig. 2, the cumulative survival rate of patients with a TS of 4.4–9.6 cm was significantly lower than those in the < 4.4 cm group, and it was the lowest in the > 9.6 cm group. In the COX risk regression model, a larger TS was positively associated with a higher risk of mortality, and HRs (95%CI) in the 4.4–9.6 cm and > 9.6 cm groups were 1.64 (1.53–1.76) and 2.32 (2.10–2.56) compared with the < 4.4 cm group, respectively (*P* for Trend: < 0.001), indicating that an increased TS in GSRC patients was significantly associated with a higher risk of death. Even in Model II and Model III, which were adjusted for age, race, marriage, primary site, grade, stage, surgery, radiotherapy and chemotherapy, positive/negative lymph nodes, and the number of tumors, this correlation was still statistically significant, as shown in Table 3.

**Table 2:** Demographic and tumor characteristics at baseline stratified by tumor size

Characteristics	level	Overall (%)	< 4.4 cm (%)	4.4–9.6 cm (%)	> 9.6 cm (%)	*P *value
		4744	2361	1817	566	
Age (years), *n* (%)	< 65	2690 (56.7)	1300 (55.1)	1061 (58.4)	329 (58.1)	0.075
	> = 65	2054 (43.3)	1061 (44.9)	756 (41.6)	237 (41.9)	
Marriage, *n* (%)	Unknown	1715 (36.2)	879 (37.2)	636 (35.0)	200 (35.3)	0.291
	Married	2858 (60.2)	1389 (58.8)	1119 (61.6)	350 (61.8)	
	Unmarried	171 (3.6)	93 (3.9)	62 (3.4)	16 (2.8)	
Sex, *n* (%)	Male	2508 (52.9)	1201 (50.9)	1012 (55.7)	295 (52.1)	0.008*
	Female	2236 (47.1)	1160 (49.1)	805 (44.3)	271 (47.9)	
Race, *n* (%)	White	3258 (68.7)	1604 (67.9)	1262 (69.5)	392 (69.3)	0.549
	Other^a^	1486 (31.3)	757 (32.1)	555 (30.5)	174 (30.7)	
Primary site, *n* (%)	proximal third^b^	1043 (22.0)	519 (22.0)	419 (23.1)	105 (18.6)	< 0.001*
	middle^c^	1437 (30.3)	803 (34.0)	500 (27.5)	134 (23.7)	
	distal third^d^	1417 (29.9)	708 (30.0)	604 (33.2)	105 (18.6)	
	Other^e^	847 (17.9)	331 (14.0)	294 (16.2)	222 (39.2)	
Grade, *n* (%)	I/II	130 (2.7)	78 (3.3)	45 (2.5)	7 (1.2)	0.029*
	III	4009 (84.5)	1971 (83.5)	1541 (84.8)	497 (87.8)	
	IV	141 (3.0)	66 (2.8)	63 (3.5)	12 (2.1)	
	Unknown	464 (9.8)	246 (10.4)	168 (9.2)	50 (8.8)	
SEER stage, *n* (%)	Distant	1236 (26.1)	477 (20.2)	567 (31.2)	192 (33.9)	< 0.001*
	Localized	1056 (22.3)	855 (36.2)	172 (9.5)	29 (5.1)	
	Regional	2380 (50.2)	981 (41.6)	1060 (58.3)	339 (59.9)	
	Unknown	72 (1.5)	48 (2.0)	18 (1.0)	6 (1.1)	
Surgery, *n* (%)	No	1164 (24.5)	593 (25.1)	460 (25.3)	111 (19.6)	0.015*
	Yes	3580 (75.5)	1768 (74.9)	1357 (74.7)	455 (80.4)	
Radiation, *n* (%)	No	3137 (66.1)	1655 (70.1)	1094 (60.2)	388 (68.6)	< 0.001*
	Yes	1607 (33.9)	706 (29.9)	723 (39.8)	178 (31.4)	
Chemotherapy, *n* (%)	No	1847 (38.9)	1097 (46.5)	568 (31.3)	182 (32.2)	< 0.001*
	Yes	2897 (61.1)	1264 (53.5)	1249 (68.7)	384 (67.8)	
Regional lymph node status, *n* (%)	Negative	1142 (24.1)	855 (36.2)	240 (13.2)	47 (8.3)	< 0.001*
	Positive	2320 (48.9)	830 (35.2)	1095 (60.3)	395 (69.8)	
	Unknown	1282 (27.0)	676 (28.6)	482 (26.5)	124 (21.9)	
Total number, *n* (%)	1	4396 (92.7)	2160 (91.5)	1692 (93.1)	544 (96.1)	< 0.001*
	> 1	348 (7.3)	201 (8.5)	125 (6.9)	22 (3.9)	
Vital status (%)	Alive	1162 (24.5)	804 (34.1)	53 (9.4)	305 (16.8)	< 0.001*
	Dead	3582 (75.5)	1557 (65.9)	513 (90.6)	1512 (83.2)	
Survival time (month)		37.9 (42.7)	47.3 (46.7)	31.0 (37.62)	20.9 (29.1)	< 0.001*

SEER stage, SEER stage is defined by the SEER database referred to as the SEER stage in our article that provides information about each cancer (primary site/histology/other factors defined) schema

*SEER* surveillance, epidemiology, and end results program

^a^Other, American Indian/Alaska Native/Asian or Pacific Islander/Black/Unknown

^b^proximal third, cardia/fundus

^c^middle, body/lesser curvature /greater curvature

^d^distal third, antrum/pylorus

^e^Other, NOS/overlapping lesion

^*^P < 0.05

**Table 3:** Cox risk proportional regression model for tumor size in GSRC patients

Variable		Model I			Model II			Model III	
	HR	95% CI	*P *value	HR	95% CI	*P *value	HR	95% CI	*P *value
Tumor size	P for Trend:^<0.001^*			P for Trend:^<0.001^*			P for Trend:^<0.001^*		
< 4.4 cm	1.0 (Reference)			1.0 (Reference)			1.0 (Reference)		
4.4–9.6 cm	1.64	1.53–1.76	< 0.001*	1.66	1.54–1.78	< 0.001*	1.25	1.16–1.35	< 0.001*
> 9.6 cm	2.32	2.10–2.56	< 0.001*	2.35	2.12–2.59	< 0.001*	1.77	1.59–1.97	< 0.001*

Model I was not adjusted

Model II was adjusted for age, race, and marriage

Model III was further adjusted for the primary site, grade, SEER stage, surgery, radiotherapy and chemotherapy, regional lymph node status, and the number of tumors on the basis of model II

*HR* hazard ratio, *95% CI* 95% confidence interval, *GSRC* gastric signet ring cell carcinoma

^*^P < 0.05

### Independent predictors for patients with GSRC

According to univariate analysis, age, marriage, race, primary site, grade, SEER stage, surgery, radiation, chemotherapy, regional lymph node status, TS, and total number were PFs for OS. According to multivariate analysis of these variables, age (< 65 years as a reference; ≥ 65 years: HR 1.41, 95%CI 1.30–1.53), marriage (unknown as a reference; married: HR 0.85, 95% CI 0.78–0.93; unmarried: HR 0.87, 95% CI 0.70–1.09), chemotherapy (No as a reference; Yes: HR 0.63, 95% CI 0.57–0.69), surgery (Yes as a reference; No: HR 1.94, 95% CI 1.58–2.38), SEER stage (distant as a reference; localized: HR 0.24, 95% CI 0.20–0.29; regional: HR 0.53, 95% CI 0.47–0.59), regional lymph node status (negative as a reference; positive: HR 1.73, 95%CI 1.49–2.02), total number (1 as a reference; > 1: HR 0.71, 95% CI 0.61–0.83), and TS (< 4.4 cm as a reference; 4.4–9.6 cm: HR1.31, 95% CI 1.20–1.44; > 9.6 cm: HR 1.95, 95% CI 1.71–2.22) were significant independent PFs for OS. (Table 4).

**Table 4:** Univariate and multivariable analysis of factors associated with overall survival in GSRC patients

Variables	level	Univariate analysis	*P *value	Multivariate analysis	*P *value
		HR (95% CI)		HR (95% CI)	
Age (years)	< 65	Reference		Reference	
	> = 65	1.37(1.26–1.48)	*P* < 0.001*	1.41(1.30–1.53)	*P* < 0.001*
Marriage	Unknown	Reference		Reference	
	Married	0.82(0.76–0.89)	*P* < 0.001*	0.85(0.78–0.93)	0.001*
	Unmarried	0.8(0.64–0.99)	0.045*	0.87(0.70–1.09)	0.232
Sex	Male	Reference		Reference	
	Female	0.97(0.89–1.05)	0.403	*-*	*-*
Race	White	Reference		Reference	
	Other^a^	0.79(0.72–0.86)	*P* < 0.001*	0.93(0.85–1.02)	0.109
Primary site	proximal third^b^	Reference		Reference	
	middle^c^	0.66(0.6–0.74)	*P* < 0.001*	0.9(0.80–1.01)	0.061
	distal third^d^	0.69(0.62–0.77)	*P* < 0.001*	0.99(0.88–1.11)	0.871
	Other^e^	0.93(0.83–1.05)	0.267	1.07(0.94–1.21)	0.324
Grade	I/II	Reference		Reference	
	III	1.01(0.80–1.27)	0.921	0.95(0.75–1.2)	0.665
	IV	1.1(0.80–1.52)	0.543	1.12(0.81–1.54)	0.483
	Unknown	1.41(1.09–1.82)	0.009*	0.87(0.67–1.12)	0.275
SEER stage	Distant	Reference		Reference	
	Localized	0.14(0.12–0.16)	*P* < 0.001*	0.24(0.20–0.29)	P < 0.001*
	Regional	0.35(0.32–0.38)	*P* < 0.001*	0.53(0.47–0.59)	P < 0.001*
	Unknown	0.74(0.56–0.98)	0.038*	0.39(0.29–0.53)	P < 0.001*
Tumor size (cm)	< 4.4	Reference		Reference	
	4.4–9.6	1.71(1.57–1.86)	*P* < 0.001*	1.31(1.20–1.44)	P < 0.001*
	> 9.6	2.48(2.19–2.80)	*P* < 0.001*	1.95(1.71–2.22)	P < 0.001*
Surgery	No	3.61(3.30–3.94)	*P* < 0.001*	1.94(1.58–2.38)	P < 0.001*
	Yes	Reference		Reference	
Radiation	No	Reference		Reference	
	Yes	0.87(0.80–0.94)	*P* < 0.001*	0.97(0.88–1.07)	0.565
Chemotherapy	No	Reference		Reference	
	Yes	1.13(1.05–1.23)	0.003*	0.63(0.57–0.69)	P < 0.001*
Regional lymph node status	Negative	Reference		Reference	
	Positive	2.95(2.62–3.31)	*P* < 0.001*	1.73(1.49–2.02)	P < 0.001*
	Other	6.56(5.78–7.45)	*P* < 0.001*	2.42(1.94–3.00)	P < 0.001*
Total_number	1	Reference		Reference	
	> 1	0.65(0.55–0.75)	*P* < 0.001*	0.71(0.61–0.83)	P < 0.001*

SEER stage, SEER stage is defined by the SEER database referred to as the SEER stage in our article that provides information about each cancer (primary site/histology/other factors defined) schema

*SEER* surveillance, epidemiology, and end results program, *HR* hazard ratio, *95% CI,* 95% confidence interval, *GSRC* gastric signet ring cell carcinoma

^a^Other, American Indian/Alaska Native/Asian or Pacific Islander/Black/Unknown;

^b^proximal third, cardia/fundus;

^c^middle, body/lesser curvature /greater curvature;

^d^distal third, antrum/pylorus;

^e^Other, NOS/overlapping lesion;

^*^P < 0.05

### Nomogram construction

**Fig. 3:**
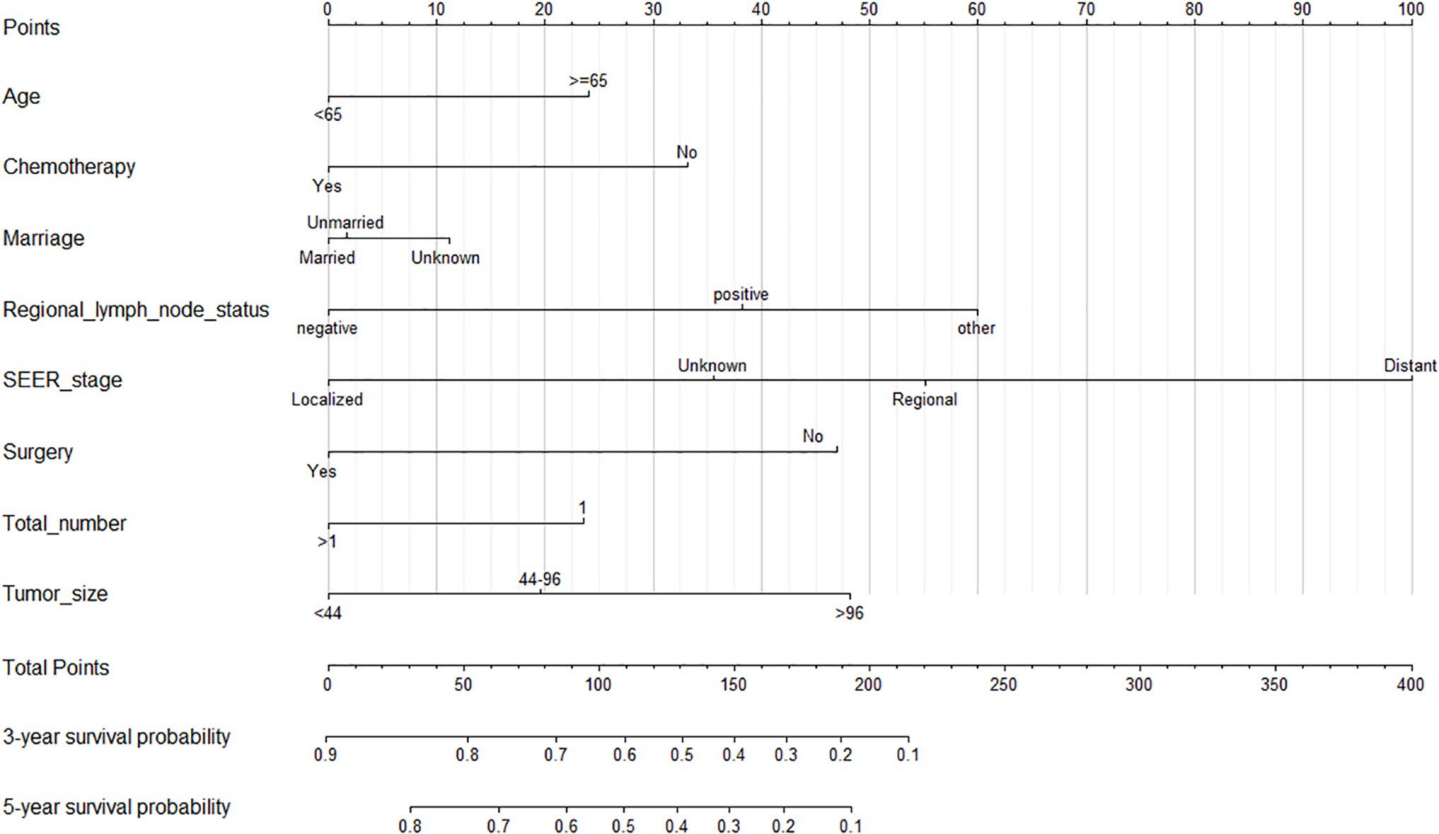
3-and 5-year OS prediction nomogram for GSRC patients. *OS* overall survival; *GSRC* gastric signet ring cell carcinoma

**Fig. 4:**
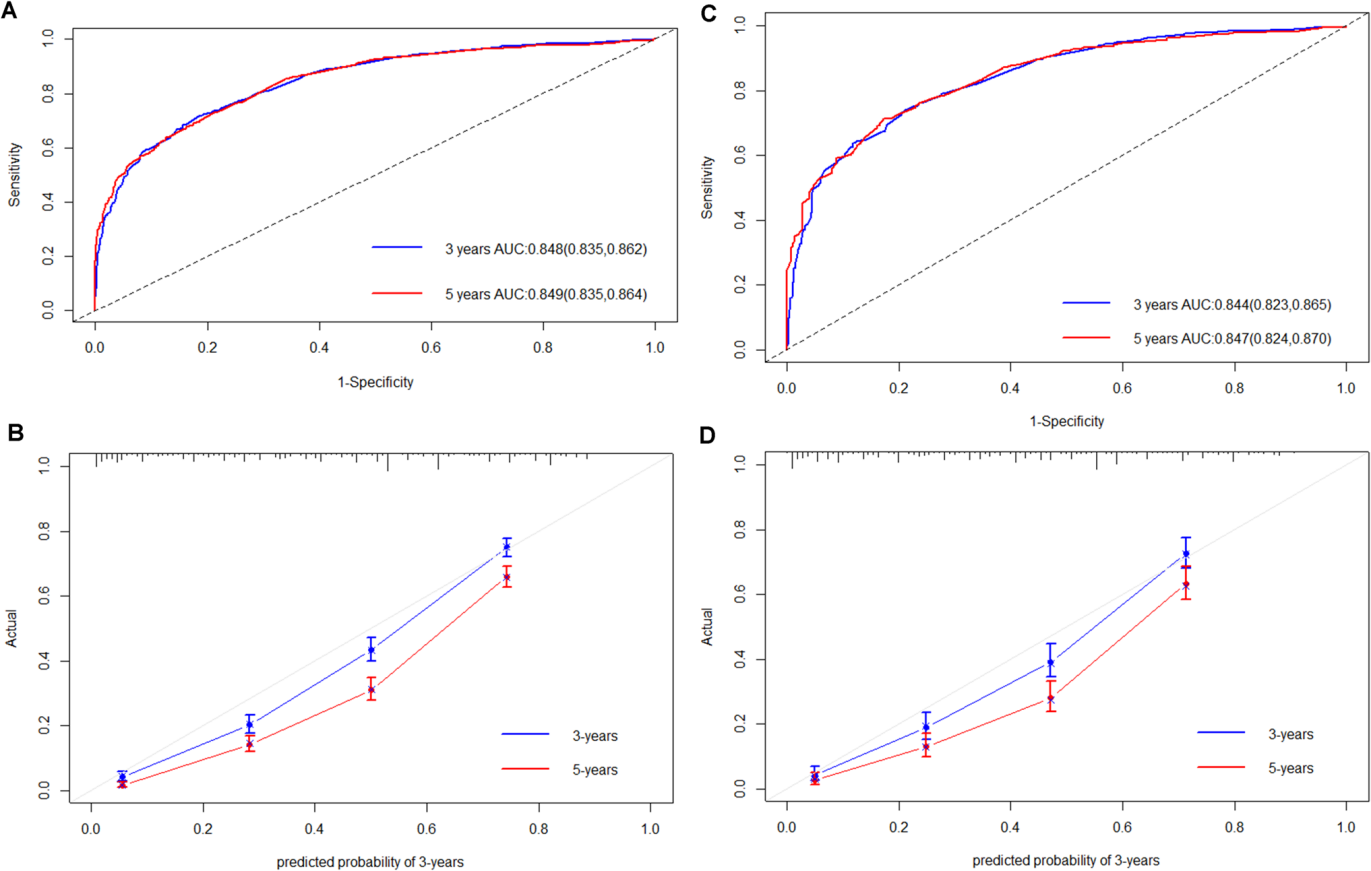
ROC curves and Calibration curves of 3- and 5-year OS prediction nomograms for GSRC patients. ROC curves (**A** Training set, **B** Validation set); Calibration curves (**C** Training set, **D** Validation set). *OS* overall survival, *GSRC* gastric signet ring cell carcinoma, *ROC* receiver operating characteristic, *AUC* area under receiver operating characteristic curve

Based on independent PFs obtained through multivariate COX regression analysis, a nomogram was constructed to predict 3- and 5-year OS in GSRC patients (Fig. 3). The C index of the nomogram was 0.748 (95% CI 0.739–0.758) in the training set and 0.745 (95% CI 0.730–0.760) in the validation set, all greater than 0.7, indicating favorable accuracy. In addition, the AUC values for 3- and 5-year were 0.848 (95% CI 0.835–0.862) and 0.849 (95% CI 0.835–0.864) in the training set, and 0.844 (95% CI 0.823–0.865) and 0.847 (95% CI 0.824–0.870) (Fig. 4A, B) in the validation set respectively. The calibration curve indicates that our nomogram is very close to an ideal curve. (Fig. 4C, D) These results show that our model has favorable accuracy in both the training set and the validation set.

## Discussion

TS, which can be measured objectively, has been incorporated into prognostic models for several malignancies, such as lung cancer, breast cancer, and liver cancer [11–15]. However, according to the current TNM staging system, the T stage of GC is defined as the depth of tumor invasion instead of the TS[16]. The prognostic significance of TS for patients with GC remains controversial. Recently, Xiao et al. classified TS into: small (≤ 2.5 cm), medium (2.6–5.2 cm), and large (≥ 5.3 cm). Their study indicated that the increase in TS had no independent negative impact on the prognosis[17]. In addition, in the study of Yokota et al., the patients were assigned to three subgroups with 2 cm and 7 cm as the boundaries. According to the univariate analysis results, TS was clinically a predictive marker for the survival of patients with GC, while in multivariate analysis, positive LNM, depth of invasion, and tumor location were more important than TS[18]. However, many studies have indicated that TS has an association with the depth of invasion and LNM, and is also an independent PF for patients with GC. Xiaowen Liu et al. divided the TS into two groups (≥ 6 cm, < 6 cm), and they found a significant difference in the survival rate between the two groups[19]. Wang et al. divided the maximum tumor diameter into four subgroups (≤ 2, ≤ 3, ≤ 5, > 5 cm), and the results showed that TS was an independent PF for GC[20]. Xu et al. assigned the patients to two groups with a threshold of 4.75 cm, and their study also obtained similar results[21]. These findings suggest that TS is of great value in predicting the prognosis of GC patients, but it is difficult to reach a consensus on an optimal cut-off value of TS for GC worldwide.

GSRC features broad clinical behaviors and is a subtype of GC with a higher degree of malignancy. The risk stratification of patients with GSRC is rarely investigated. Liyuan Zhou et al. performed a retrospective analysis of the clinical data on 946 patients with GSRC, and found that TS was an independent RF affecting the prognosis of the patients. GSRC patients with larger tumors are at a higher risk for invasive growth, LNM, and distant metastasis. In this study, the cut-off value of TS was determined as 4.9 cm without interpretation (using X-tile)[22]. Most studies used X-tile or median to determine the cut-off value. In this case, the relationship between continuous variables and the survival of patients was linear by default, ignoring the possible non-linear relationship between them. Therefore, the cut-off value could not truly reflect the relationship between TS and the survival of the patients. In view of the importance of TS on the prognosis of patients, the optimal cut-off value of TS that affects the prognosis of GSRC patients was determined based on RCS in the present study. At present, RCS is widely used to describe the non-linear relationship between variables. For example, Huolun Feng et al. used the RCS model to visualize the correlation between TS and the prognosis of patients with colon cancer, and found a significant non-linear relationship between TS and the prognosis of patients. Compared with patients with TS > 4 cm, the mortality risk of patients with TS < 4 cm has a sudden increase, and overall, patients with larger TS have a worse prognosis[23]. A study involving 5424 patients demonstrated a significant association between TS and the rate of pancreatic neuroendocrine tumor metastasis, and the likelihood of metastasis increased in a non-linear manner with increasing TS. TS ≤ 2 cm was an independent influencing factor for better prognosis[24]. To our knowledge, this paper is the first to explore the non-linear relationship between TS and the all-cause mortality risk of GSRC patients based on the RCS model.

This study included 4744 GSRC patients to analyze the TS cut-off value that affected the prognosis of GSRC patients, and further combined with other prognostic factors to predict the OS of GSRC patients. Our research reveals that in the constructed RCS model, as TS increases, the risk of mortality in patients elevates accordingly. When TS is 4.4 cm, the corresponding HR is close to 1. As TS continues to increase in the range of 4.4–9.6 cm, the risk of mortality in patients increases significantly and keeps rising. When TS exceeds 9.6 cm, the risk of death gradually plateaus out but remains at a high level. Hence, TSs at 4.4 cm and 9.6 cm were used to group the patients. The KM survival analysis showed significant inter-group differences in the log-rank test results of the three groups of patients. The cumulative survival rate of patients in the 4.4–9.6 cm group was significantly lower than that of patients in the < 4.4 cm group, and was the lowest in the > 9.6 cm group. Cox regression analysis results showed that a larger TS was significantly associated with a higher risk of death. This suggests that TS is an independent prognostic factor in patients with GSRC. The study by Qing Wei, et al.[25] also reported similar findings. To elucidate the reason why larger tumors have worse prognosis in GSRC patients, many studies have been conducted. For instance, Hiroaki Saito et al. found that among all large tumors (> 8 cm), 71.8% were undifferentiated with higher T and N stages, and their incidence of peritoneal recurrence was also higher than that in patients with small tumors [26].

These findings revealed that GSRC patients with larger tumors might have a higher risk of invasive growth, lymphatic node metastasis LNM, and distant metastasis with peritoneal dissemination [22]. This may suggest that because of the unique biological behavior of the GSRC, patients with large larger tumors (> 4.4 cm), regardless of the T stage and N stage, should be given more aggressive clinical treatment, such as early radical surgical resection, chemoradiotherapy and immunotherapy, and an aggressive follow-up strategy. In addition to TS, this study also revealed that OS was closely related to age, marriage, chemotherapy, surgery, SEER stage, regional lymph node status, and total number. Studies have shown that the clinicopathological features of early and advanced GSRC are quite different. Early GSRC had a better prognosis than other kinds of GC, while the prognosis of advanced GSRC was worse than that of other kinds of GC [27]. Progressive GSRC may feature more obvious infiltration and is more prone to LNM [28].

To facilitate clinicians evaluate the 3-year and 5-year all-cause mortality risk of GSRC patients, we further constructed a nomogram prediction model based on eight indicators including TS. In this study, C index, ROC curve analysis, and calibration curve showed relatively good results in both the training set and the validation set, indicating that the nomogram can accurately predict the OS of GSRC patients.

There are some limitations in this study. On the one hand, the present study was retrospective with data obtained from public data records. Since the SEER database did not include detailed information about endoscopy, imaging, and peritoneal metastasis, the cut-off value (4.4 cm and 9.6cm) identified in this study needs to be verified by multicenter data in future. On the other hand, further subgroup analyses are needed in future to clarify the prognostic value of TS at different T stages.

## Conclusion

TS has significant prognostic effects for patients with GSRC. There was a non-linear relationship between TS and OS in GSRC patients, with the thresholds of TS at 4.4 cm and 9.6 cm. TS of “4.4–9.6 cm” and “ > 9.6 cm” was significantly associated with a higher risk of death compared to TS of “ < 4.4 cm”. These findings can provide a reference for risk stratification of patients. The TS-stratified nomogram we constructed and validated has strong accuracy and calibration precision, and may be helpful in predicting OS of patients with GSRC.

## Data Availability

The data that support the findings of this study are available from the corresponding author upon reasonable request.

## References

[1] Sung H, Ferlay J, Siegel RL et al (2021) Global cancer statistics 2020: globocan estimates of incidence and mortality worldwide for 36 cancers in 185 countries. CA Cancer J Clin 71(3):209–249. 10.3322/caac.21660 33538338 10.3322/caac.21660

[2] Henson DE, Dittus C, Younes M et al (2004) Differential trends in the intestinal and diffuse types of gastric carcinoma in the United States, 1973–2000: increase in the signet ring cell type. Arch Pathol Lab Med 128(7):765–770. 10.5858/2004-128-765-dtitia 15214826 10.5858/2004-128-765-DTITIA

[3] Taghavi S, Jayarajan SN, Davey A et al (2012) Prognostic significance of signet ring gastric cancer. J Clin Oncol 30(28):3493–3498. 10.1200/jco.2012.42.6635 22927530 10.1200/JCO.2012.42.6635 PMC3454770

[4] Bamboat ZM, Tang LH, Vinuela E et al (2014) Stage-stratified prognosis of signet ring cell histology in patients undergoing curative resection for gastric adenocarcinoma. Ann Surg Oncol 21(5):1678–1685. 10.1245/s10434-013-3466-8 24394986 10.1245/s10434-013-3466-8

[5] Fléjou JF (2011) WHO classification of digestive tumors: the fourth edition. Ann Pathol 31(5 Suppl):S27-31. 10.1016/j.annpat.2011.08.001 22054452 10.1016/j.annpat.2011.08.001

[6] Arai T (2019) Where does signet-ring cell carcinoma come from and where does it go? Gastric Cancer 22(4):651–652. 10.1007/s10120-019-00960-w 30963318 10.1007/s10120-019-00960-w

[7] Japanese Gastric Cancer A (1998) Japanese classification of gastric carcinoma—2nd english edition. Gastric Cancer 1(1):10–24. 10.1007/s101209800016 11957040 10.1007/s101209800016

[8] Lauren P (1965) The two histological main types of gastric carcinoma: diffuse and so-called intestinal-type carcinoma: an attempt at a histo-clinical classification. Acta Pathol Microbiol Scand 64:31–49. 10.1111/apm.1965.64.1.31 14320675 10.1111/apm.1965.64.1.31

[9] Piessen G, Messager M, Leteurtre E et al (2009) Signet ring cell histology is an independent predictor of poor prognosis in gastric adenocarcinoma regardless of tumoral clinical presentation. Ann Surg 250(6):878–887. 10.1097/SLA.0b013e3181b21c7b 19855261 10.1097/SLA.0b013e3181b21c7b

[10] Li C, Kim S, Lai JF et al (2007) Advanced gastric carcinoma with signet ring cell histology. Oncology 72(1–2):64–68. 10.1159/000111096 18004078 10.1159/000111096

[11] Liu Y, He M, Zuo WJ et al (2021) Tumor size still impacts prognosis in breast cancer with extensive nodal involvement. Front Oncol 11:585613. 10.3389/fonc.2021.585613 33898305 10.3389/fonc.2021.585613 PMC8064390

[12] Carter CL, Allen C, Henson DE (1989) Relation of tumor size, lymph node status, and survival in 24,740 breast cancer cases. Cancer 63(1):181–187. 10.1002/1097-0142(19890101)63:1%3c181::aid-cncr2820630129%3e3.0.co;2-h 2910416 10.1002/1097-0142(19890101)63:1<181::aid-cncr2820630129>3.0.co;2-h

[13] Fukui T, Fukumoto K, Okasaka T et al (2016) Prognostic impact of tumour size in completely resected thymic epithelial tumours. Eur J Cardiothorac Surg 50(6):1068–1074. 10.1093/ejcts/ezw178 27999073 10.1093/ejcts/ezw178

[14] Yamamoto Y, Okamura Y, Uemura S et al (2017) Vascularity and tumor size are significant predictors for recurrence after resection of a pancreatic neuroendocrine tumor. Ann Surg Oncol 24(8):2363–2370. 10.1245/s10434-017-5823-5 28271173 10.1245/s10434-017-5823-5

[15] Zhang Y, Sun Y, Chen H (2016) Effect of tumor size on prognosis of node-negative lung cancer with sufficient lymph node examination and no disease extension. Onco Targets Ther 9:649–653. 10.2147/ott.S98509 26917972 10.2147/OTT.S98509 PMC4751902

[16] Amin MB, Greene FL, Edge SB et al (2017) The eighth edition AJCC cancer staging manual: continuing to build a bridge from a population-based to a more “personalized” approach to cancer staging. CA Cancer J Clin 67(2):93–99. 10.3322/caac.21388 28094848 10.3322/caac.21388

[17] Xiao J, Shen K, Fan H et al (2023) Prognostic value of tumor size in gastric cancer: a retrospective cohort study based on SEER database. Int J Surg Pathol. 10.1177/10668969231152578 36802927 10.1177/10668969231152578

[18] Yokota T, Ishiyama S, Saito T et al (2002) Is tumor size a prognostic indicator for gastric carcinoma? Anticancer Res 22(6b):3673–3677 12552975

[19] Liu X, Xu Y, Long Z et al (2009) Prognostic significance of tumor size in T3 gastric cancer. Ann Surg Oncol 16(7):1875–1882. 10.1245/s10434-009-0449-x 19373514 10.1245/s10434-009-0449-x

[20] Wang X, Wan F, Pan J et al (2008) Tumor size: a non-neglectable independent prognostic factor for gastric cancer. J Surg Oncol 97(3):236–240. 10.1002/jso.20951 18095266 10.1002/jso.20951

[21] Xu M, Huang CM, Zheng CH et al (2014) Does tumor size improve the accuracy of prognostic predictions in node-negative gastric cancer (pT1-4aN0M0 stage)? PLoS ONE 9(7):e101061. 10.1371/journal.pone.0101061 25003849 10.1371/journal.pone.0101061 PMC4086925

[22] Zhou L, Li W, Cai S et al (2019) Large tumor size is a poor prognostic factor of gastric cancer with signet ring cell: results from the surveillance, epidemiology, and end results database. Medicine (Baltimore) 98(40):e17367. 10.1097/md.0000000000017367 31577736 10.1097/MD.0000000000017367 PMC6783183

[23] Feng H, Lyu Z, Zheng J et al (2021) Association of tumor size with prognosis in colon cancer: a surveillance, epidemiology, and end results (SEER) database analysis. Surgery 169(5):1116–1123. 10.1016/j.surg.2020.11.011 33334582 10.1016/j.surg.2020.11.011

[24] Liu Y, Ye S, Zhu Y et al (2019) Impact of tumour size on metastasis and survival in patients with pancreatic neuroendocrine tumours (PNETs): a population based study. J Cancer 10(25):6349–6357. 10.7150/jca.27779 31772667 10.7150/jca.27779 PMC6856747

[25] Wei Q, Gao Y, Qi C et al (2021) Clinicopathological characteristics and prognosis of signet ring gastric cancer: a population-based study. Front Oncol 11:580545. 10.3389/fonc.2021.580545 34490073 10.3389/fonc.2021.580545 PMC8418067

[26] Saito H, Osaki T, Murakami D et al (2006) Macroscopic tumor size as a simple prognostic indicator in patients with gastric cancer. Am J Surg 192(3):296–300. 10.1016/j.amjsurg.2006.03.004 16920421 10.1016/j.amjsurg.2006.03.004

[27] Chon HJ, Hyung WJ, Kim C et al (2017) Differential prognostic implications of gastric signet ring cell carcinoma: stage adjusted analysis from a single high-volume center in asia. Ann Surg 265(5):946–953. 10.1097/sla.0000000000001793 27232252 10.1097/SLA.0000000000001793 PMC5389602

[28] Kao YC, Fang WL, Wang RF et al (2019) Clinicopathological differences in signet ring cell adenocarcinoma between early and advanced gastric cancer. Gastric Cancer 22(2):255–263. 10.1007/s10120-018-0860-8 30069742 10.1007/s10120-018-0860-8

